# Mitigation of jet cross-flow induced vibrations using an innovative biomimetic nozzle design inspired by shark gill geometry

**DOI:** 10.1038/s41598-022-15026-8

**Published:** 2022-06-30

**Authors:** Ibrahim Gad-el-Hak, Njuki Mureithi

**Affiliations:** Department of Mechanical Engineering, Polytechnique Montréal, Montreal, QC H3C 3A7 Canada

**Keywords:** Zoology, Engineering

## Abstract

Shark gill slits enable sharks to eject the water after the oxygen has been removed in ram ventilation. The reduced effect of jet flow from the gill slits gives sharks smooth maneuverability. A biomimetic “shark nozzle” is proposed to improve mixing between the jet flow and surrounding fluid. Jet flow systems are an essential component of many industrial applications. The key characteristic of jet flow is the mixing process that occurs between two fluid streams to allow heat and/or mass transfer between them. Many industrial and propulsion devices that use jet flows need rapid mixing for effective and environmentally friendly operation. Fuel-injection systems, chemical reactors, and heating and air conditioning systems are examples of devices where a mixing process takes place. Recently, jet flow plays an important role in design and operation of specialized nuclear pressurized water reactors (PWRs). The loss-of-coolant accident (LOCA) holes and slots machined in the core periphery baffle plates are designed to mitigate the effects of a severe LOCA event. However, in normal operation, the holes are a source of a jet flow that can induced vibrations in the fuel assemblies near the baffle before being mixed with the surrounding fluid. This may cause wear and fretting in fuel rods with their supports. The ultimate solution to prevent the fuel assembly vibrations from LOCA hole jetting in a reactor is enhancing mixing of a jet flow with ambient flow in order to rapidly reduce jet momentum. This work proposes a new shark-inspired nozzle design that exploits the observed high efficiency capacity of shark gill slits. Tests are conducted to evaluate the performance of the new design. The obtained results show that the shark-inspired biomimetic nozzle has a greater effect on the rod bundle vibration, and the critical velocity at which the unstable vibration occurs in the rod bundle is delayed by 20% using the biomimetic nozzle. In addition to delaying instability, a vibration amplitude reduction of 85% was obtained by using the proposed shark-inspired nozzle instead of the circular nozzle.

## Introduction

Over the last few decades, jet flows have received much attention^1–4^. A jet’s flow field can be described as follows. At the jet centerline, the flow is uniform while a shear layer is generated near the nozzle wall, which is defined as a top-hat velocity profile. As a result of generating the shear layer with thickness ($\theta$), small disturbances, normally described by their Strouhal number ($St_{\theta }$=$f\theta /U$), are amplified and eventually roll-up into a coherent structure and periodic sets of vortices, making it subject to the Kelvin-Helmholtz, or the shear-layer instability^5^. The dynamics of these vortices dominate shear layer growth, which is defined by phenomenon such as vortex pairing. The dominant jet instability mode, which is described by the Strouhal number based on the nozzle diameter ($St_{D}$=*fD*/*U*), becomes the preferred mode as the vortices grow and develop towards the end of the potential core. The interaction of vortices beyond the end of the potential core causes complex non-linear motion, which breaks the flow’s coherent structure and results in transition to turbulent flow. One of the most important aspects of a turbulent jet is its ability to entrain more fluid from its surroundings due to the presence of the shear-layer flow at the jet boundary.

Turbulent mixing is used in many industrial components to transfer heat and mass between two or more fluid streams as in gas burners, chemical reactors, liquid mixers, gas mixers, heat exchangers, and atomizers. Efficient and rapid mixing is a particularly important to the performance of these components. Passive flow control techniques for increasing the rate of jet mixing in circular nozzle flows have been investigated by a large number of researchers^6–10^. One of the most effective passive control techniques is the application of mechanical tabs at the exit plane of the nozzle. Tab-controlled jets have received serious consideration due to their practical applications. Tabs have been discovered to have a considerable impact on entrainment and jet mixing rate due to their ability to generate streamwise vortex pairs. However, the key parameters of tab-controlled jets include the effect of tab geometry, tab number, tab orientation, tab size, and tab position relative to nozzle outlet^9^. These parameters are still under investigation. The most commonly used tab in the literature is the delta tab (triangular shape). The tab number is varied from one tab to four tabs. Tab shape and number are the most important parameters that affect the mixing rate, thus they should be designed with care.

In addition to the conventional jet flow applications, some nuclear reactor vessel designs employ holes that are machined into the core baffle plates to relieve pressure differentials during a Loss of Coolant Accident (LOCA). A Loss of Coolant Accident (LOCA) is a mode of failure for reactor cores that is caused by a break in the reactor coolant pressure boundary. As a result of this failure scenario, the pressure difference across the baffle plates inside the reactor core is increased. During normal operation, the fluid flow though the LOCA holes has a small effect on the core average flow field, but it is a source of jet flow that can result in fretting failures of fuel assemblies, as reported by the IAEA^11^. A similar failure mode involved defective joints between baffle plates that allow high jet flows through narrow gaps. This phenomenon is called *baffle jetting* and may induce fuel rod vibrations that cause fretting and wear of the fuel cladding that contain the fission products. This type of failure has been observed since the 1970s^12^ and more recently at the North Anna power plant in 2014^13^ involving damage to two fuel rods. While baffle jetting is caused by a degradation of the baffle plate bolts, and LOCA hole flow jetting is a result of design choices, both phenomena underscore the importance of understanding jet cross-flow induced vibrations.

Current reactors have a significant difficulty in reducing the resulting vibrations in their fuel assemblies due to circular jet cross-flow. From a manufacturing prospective, a circular nozzle shape for LOCA holes was proposed as it is easier to drill holes in the baffle plates. However, the mixing rate between the jet flow and the surrounding fluid, on the other hand, is a key factor in rod bundle vibration; switching from linear momentum into angular momentum (i.e vortices) would reduce the resulting vibrations. Changes to the nozzle shape are used in passive jet control to improve mixing. It is also feasible to boost the swirl flow at the exit jet boundary and improve the flow condition at the rod bundle with appropriately designed jet nozzles. Thus, using a biomimicry design approach, a new nozzle shape inspired by shark gill slits is proposed. As a result, a LOCA hole shape with adequate mixing enhancement capabilities will represent a step forward from circular nozzles.

Nowadays and in the future, engineers and designers will do well to pay attention to specific detail and systems design in nature so as to draw inspiration for efficient, renewable, self-sustaining, and ultimate solutions to many engineering problems. Biomimicry, biomimetics, and bio-inspired design are alternative terms for an innovative approach to design and engineering, inspired by design in nature. Nature-inspired innovation can lead to new paths of exploration and problem-solving opportunities that have previously been ignored. Nature is the prime example of ‘eco-design,’ and it demands the full attention of those working on renewable energy, materials, medical engineering, and technical advancement issues. An example of applied biomimicry from the seas is humpback whales. Humpback whale flippers feature non-smooth leading edges (i.e. tubercles), which showed fluid dynamic improvements as compared to the smooth leading edges of our turbines and fans. Tubercles reduce drag by 32%, increase lift by 8%, and increase angle of attack by 40% over smooth flippers before stalling in wind tunnel testing of model humpback whale flippers with and without tubercles^14^. There is also much to learn from birds. The composite turbine blade of a turboexpander was designed using biomimicry of bird flight wings, with fibre orientations that mimicked the structure of the bird’s feathers^15^. The author^15^ found that the same barb angle inspired by the flight feather was used to produce the smallest tip deflection of the rotor blades, resulting in an 80% weight reduction of biomimetic blades over stainless steel rotor blades.

**Figure 1:**
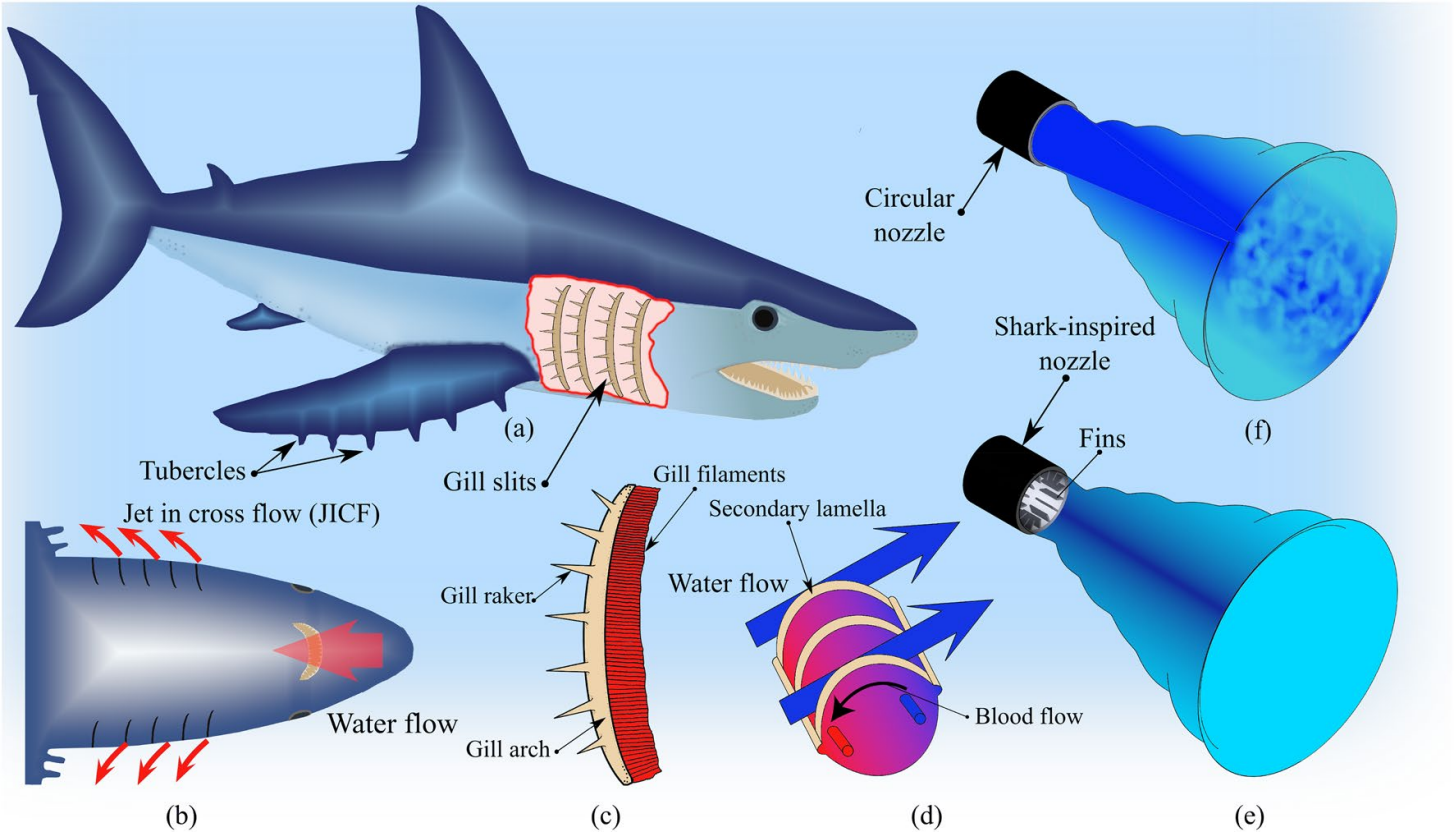
Modelling of bio-inspired nozzle. (**a**) shark anatomy, (**b**) ram ventilation concept corresponding to jet in cross flow, (**c**) drawing of a gill showing gill filaments (oxygen absorption), gill arch (supporting structure), and gill rakers, (**d**) schematic drawing of a portion of filaments and their secondary lamellae and also the countercurrent exchange system, (**e**) schematic drawing of shark-inspired nozzle, and (**f**) circular jet flow.

Inspired by sharks, a new biomimicry nozzle design is proposed as shown in Fig. 1. Efficient and rapid mixing is a key factor to mitigate vibrations from jet cross-flow in pressurized water reactor cores. Sharks breathe through a series of five to seven gill slits behind their heads on both sides of their bodies, which absorb oxygen from the water as it flows over them and is then flushed out. This respiration process, which involves forcing of water over the gills by swimming motions, is known as “*ram ventilation*”^16^. The water emanating through the gills into the ocean corresponds to jet in cross-flow (JICF) from an engineering perspective. However, this jet flow is ejected with little effect on the sharks to provide them with greater maneuvering and swimming capability. That means that the design of the gill slits enhances mixing between the jet flow and the surrounding water in the oceans. The jet velocity decays faster and the flow exits the slits with minimum effect on the shark.

Gills mainly consist of gill arches (*branchial arches*), gill rakers and gill filaments. Gill arches are a series of bony “loops” to support the gills. At their base are gill rakers, which protect the gills from mechanical damage. Each gill arch has filaments (protein structures) which perform gas exchange through a capillary network that provides a large surface area for the exchange process. In addition, the gill filaments are extended with the secondary gill lamellae to create inter-filament water channels, which help increase their surface area for gas exchange. The spacing and height of the secondary lamellae control the cross-section area of the water flow^17^. Furthermore, an efficient countercurrent gas exchange at the secondary gill lamellae further increases oxygen uptake and carbon dioxide release^18^. Gill rakers and filaments have an important effect on the fluid dynamics of the exiting water. Thus, simulating their effects in the jet flow will help us to design an efficient nozzle, and thereby eliminate or reduce the rod bundle vibrations.

The goal of the paper is to propose a biomimetic nozzle design based on the shark gill slits to develop more rapidly the mixing layer between the jet flow and the surrounding flow. Our proposed biomimicry approach is to attach equally spaced thin fins circumferentially at the circular nozzle base to enhance the mixing process by rapidly entraining the still fluid in the jet flow. The resulting jet cross-flow induced vibrations is investigated experimentally for three nozzle designs, (*i*) a basic circular nozzle, (*ii*) a shark-inspired nozzle with 10 fins, and (*iii*) a shark-inspired nozzle with 15 fins. The paper compares the ability of the proposed biomimetic nozzle to delay the critical velocity at which unstable vibrations occur and to damp the post-instability vibration amplitudes of the rod bundle. Comparison is made with the reference case of the circular nozzle. A high-speed camera is used to obtain full-field rod array vibrations by capturing a sequence of images for a specific duration with the image frame size including all rods in the bundle. An image processing algorithm determines the rod tip displacements fields by tracking the rod tip centers when the array is subjected to a specific jet velocity. The dynamical behavior of the rod bundle is obtained by increasing the jet velocity up to the unstable condition.

## Experimental design concept

### Shark-inspired nozzle design

**Figure 2:**
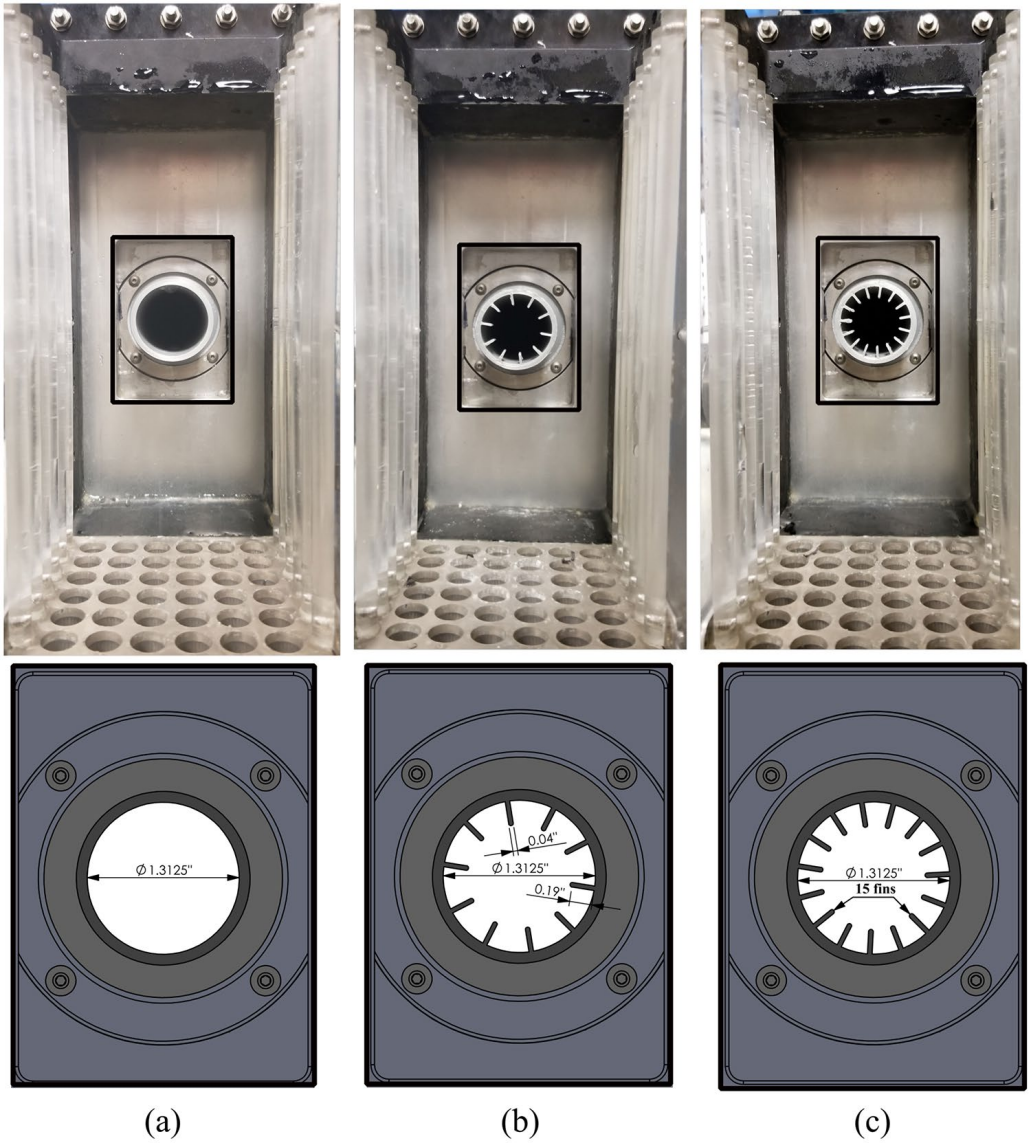
Three tested nozzle: (**a**) circular nozzle, (**b**) 10 fins shark-inspired nozzle, and (**c**) 15 fins shark-inspired nozzle.

The aim of the experiments is to look at how the rod bundle vibrates under flow conditions that cause fluid-elastic instability. Since the jet flow velocity in the test section is not constant; it is function of stream-wise and transverse direction related to jet centerline, the time-dependent displacement for each rod in the tube bundle will give us a deeper understanding about how the rods vibration is affected by the nozzle shape. Figure 2 shows the three tested nozzles, the base nozzle diameter is the same in three cases. However, very thin fins (1 mm thickness) are attached circumferentially at the circular nozzle base simulating the secondary lamella in sharks. Fin thickness should be kept to a minimum to avoid increasing the pressure drop across nozzle due to the added blockage effect. The 1 mm fin thickness is selected because it is the minimum thickness (possible for a printed nozzle) that ensured the structural integrity for fins during the testing. The fin length is 12.7 mm inside the nozzle simulating the shark’s secondary lamellae^17^. Furthermore, the effect of the number of fins on the rod bundle vibrations is investigated for two values, 10 and 15 fins.

### Experimental set up

**Figure 3:**
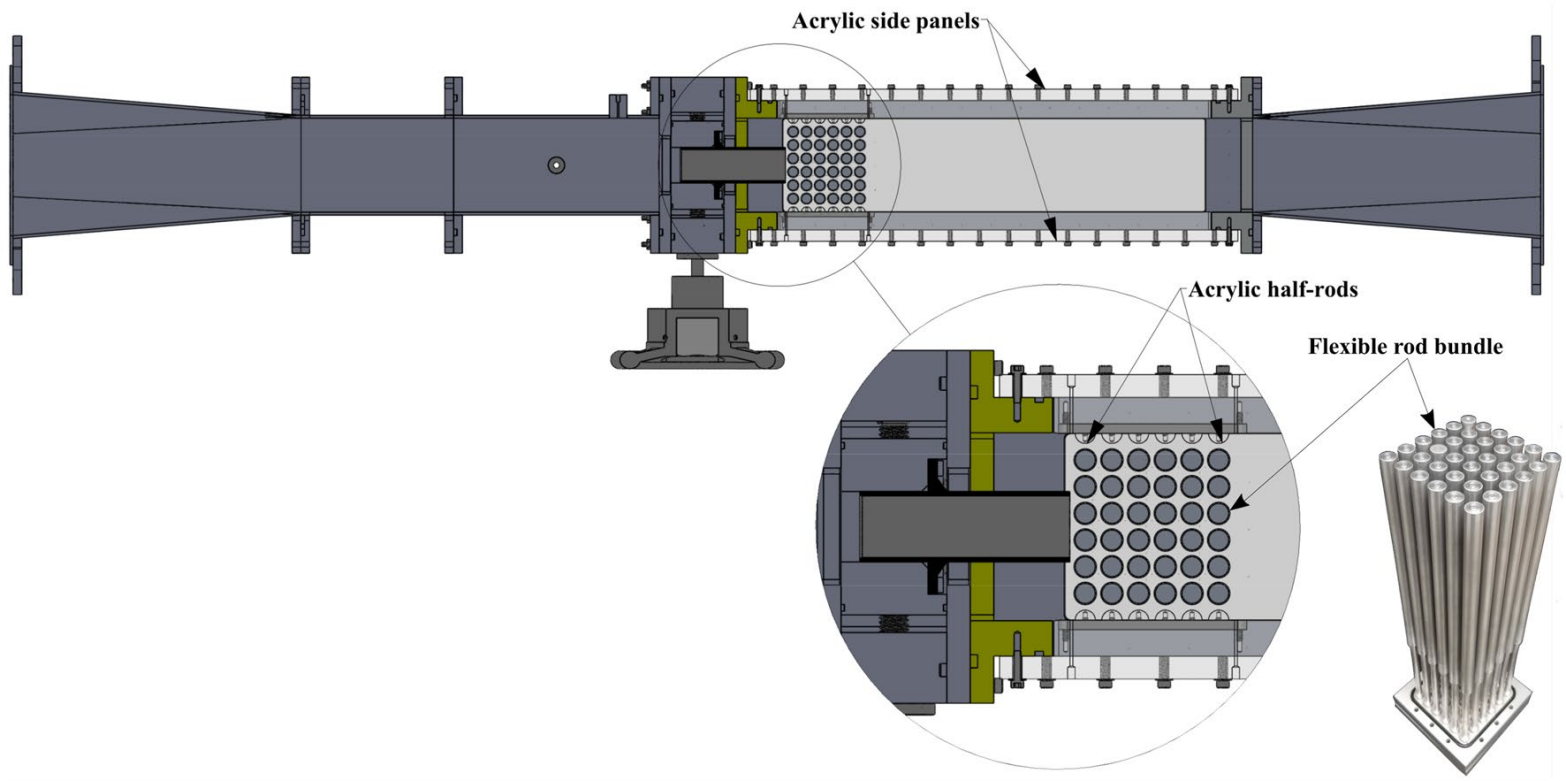
Experimental set-up.

**Figure 4:**
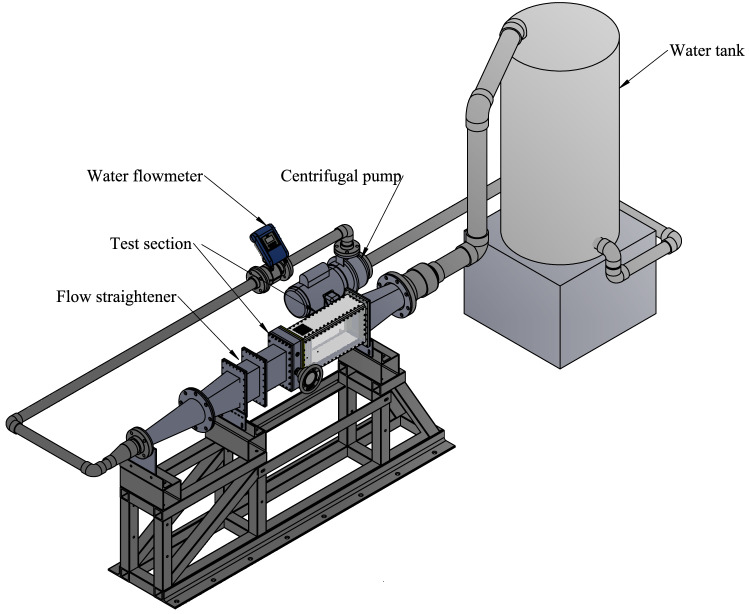
Test loop setup.

Experiments were conducted to see how the proposed biomimetic nozzle affected jet cross-flow induced vibrations. A reduced fully flexible rod bundle 6x6 is selected to investigate its dynamical behavior due to transverse jet flow. The axisymmetric rod bundle vibration has frequency of 29 Hz in still water for all rods. To form a 6x6 configuration, the flexible rods are arranged in a normal square array with a pitch-to-diameter ratio (*P*/*D*) of 1.32 as in the fuel assembly. Figure 3 shows the top sectional view of the designed tested section including the other designed components simulating what happens in the nuclear reactor cores. The uniform flow from the rectangular cross section duct is squeezed and converted to jet flow when it passes through the nozzle. The rods are exposed at their mid-span to a developed jet cross-flow. Figure 4 shows the water test loop.

### Measurement system and image processing

**Figure 5:**
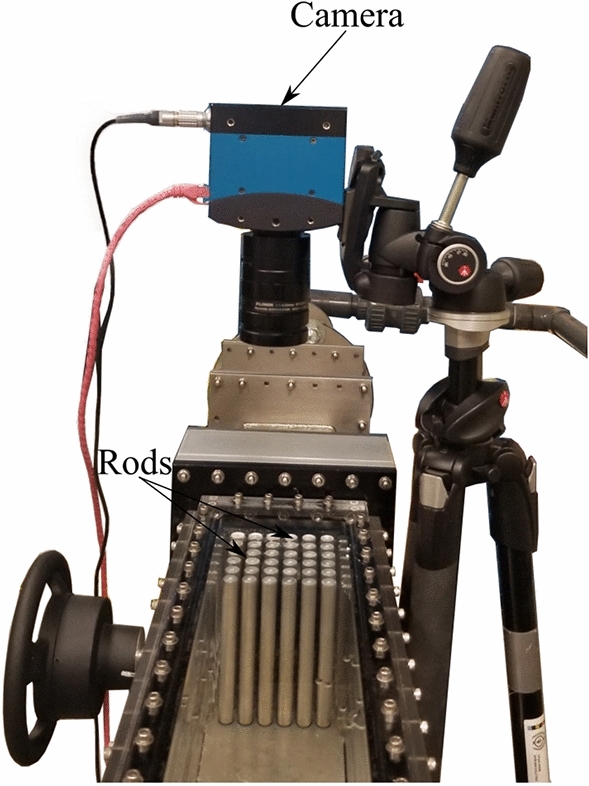
Camera set-up.

A high-speed imaging technique is considered the best solution to capture the vibration response of the rod bundle; this data acquisition method system is completely non-intrusive; no sensors (such as strain gauges) are needed on the rod surfaces, and wires are not required to be fed through the test section. Sequential images of the rod bundle vibration, during which the rods are excited by the jet cross-flow at a particular velocity, are captured using a high-speed digital camera (Motion BLITZ Cube 4, MIKROTRON). The image frame size is large enough to capture all of the rods in the tested bundle. The camera is positioned on the top panel of the test section such that its viewing plane is normal to the rods’ axis as shown in Fig. 5.

### Image processing algorithm

**Figure 6:**
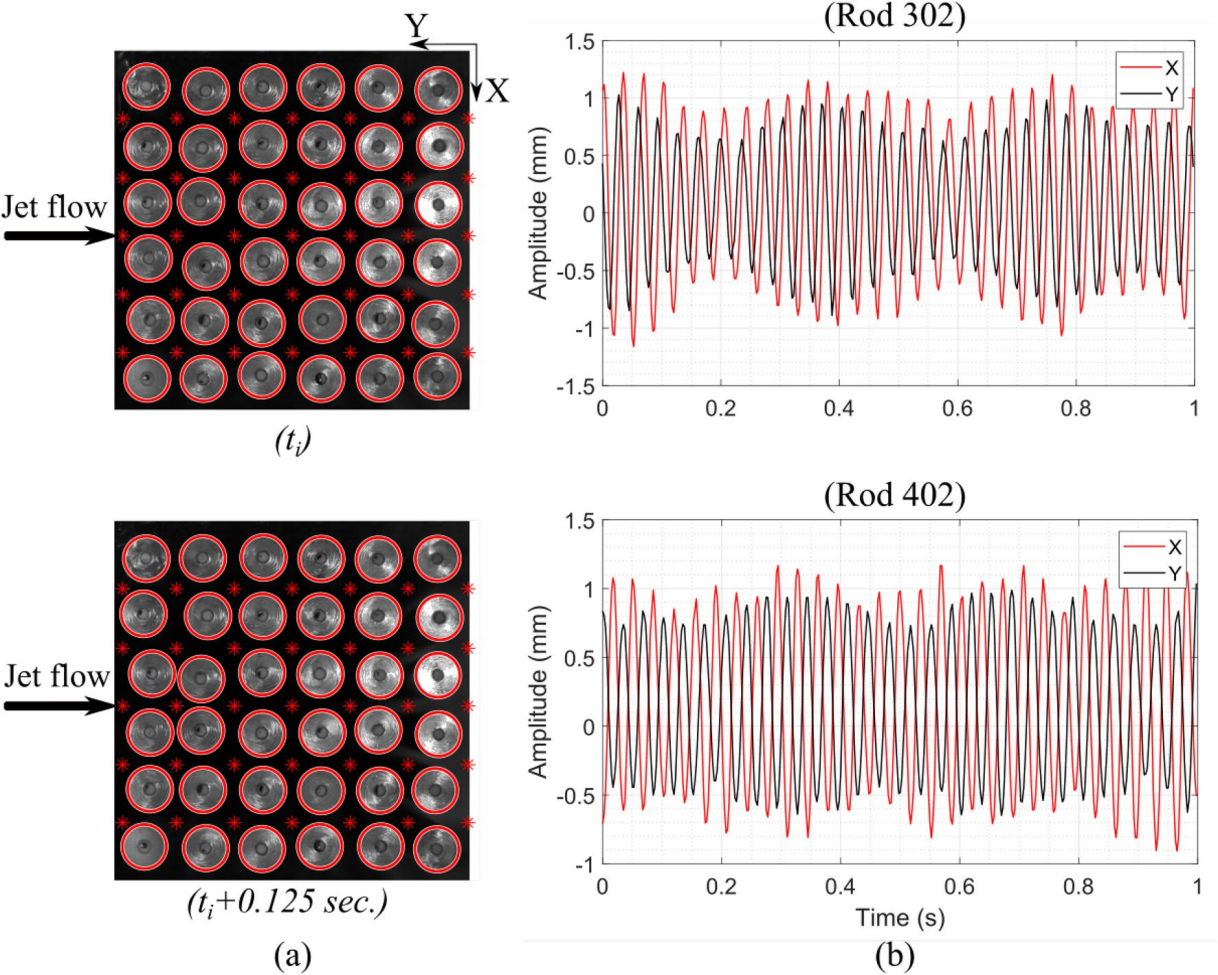
(**a**) processed images showing the detected circles by the code and the four-point boundary for each rod in the bundle, and (**b**) the time signals for two rods 302 and 402 during one second at $V_{Jet}$= 2 m/s for 15 fins shark-inspired nozzle.

To determine the rod bundle vibration at each jet flow velocity, a MATLAB image processing algorithm is generated to detect the centre of each rod in the bundle. The developed code detects the edges of rods based on the colour difference between the shiny aluminum top rod surface and the dark background test section. Thus, the image size is divided into 36 square unit cells (the rods number in the bundle), each identified by a four-point boundary in the code as shown in Fig. 6a. Figure 6 shows the vibrations that occurred for one second from rods 302 and 402. Processing 5,600 images would give us the rod bundle vibrations that occurred for 15 seconds at each velocity.

## Results

Jet cross-flow induced vibration is experimentally investigated for in-line square 6x6 rod bundle simulating a reduced fuel bundle to determine its dynamical behavior. The vibratory response of the tested bundle is obtained by increasing the jet cross-flow velocity up to the onset of an unstable vibration condition (*fluidelastic instability*). The use of a high-speed camera has the significant advantage of capturing the full-field rod bundle vibration while being completely non-intrusive. The measurements are obtained at frame-rate of 400 Hz while the rods vibrated at 29 Hz in water. The 6x6 rod bundle is first tested with the circular jet as a reference case for the comparison with the shark-inspired nozzles to show their effects on the rod bundle vibration.

### Circular nozzle jet cross- flow induced vibrations of rod bundle

**Figure 7:**
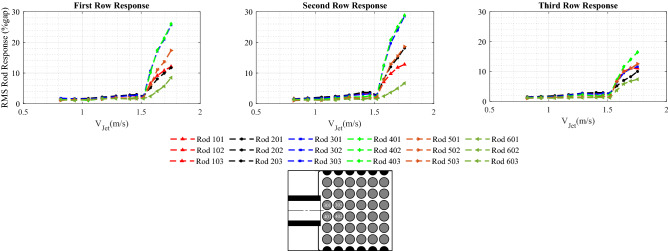
Rod bundle vibrations using the circular nozzle.

**Figure 8:**
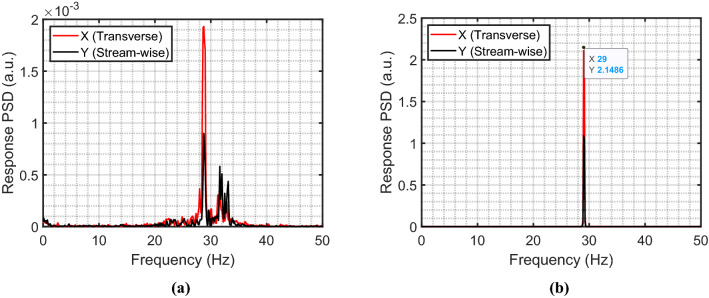
(**a**) PSD plot for Rod 401 at $V_{Jet}$ = 1.45 m/s, and (**b**) PSD plot for Rod 401 at $V_{Jet}$ = 1.75 m/s.

Figure 7 illustrates the behavior of the rod bundle subjected to the circular jet cross-flow. The RMS vibration response normalized by the inter-rod gap (*gap=Pitch-rod diameter*) is obtained in a jet velocity range from 0.93 to 1.75 m/s for each rod in the bundle. The rod responses for rows 1-3 are arranged in Fig. 7 by three sub-figures, each one included the six rods in the same row. The rods in the bundle are identified by their column number followed by their row number. For example, rod 502 is located in column number 5 and row number 2. The row number is in the direction perpendicular to the jet flow. The dynamical behavior of the rod bundle can be divided into two regions based on the jet velocity: (*i*) $V_{Jet}$ from 0.93 to 1.5 m/s and (*ii*) $V_{Jet}$ from 1.5 to 1.75 m/s. In the first region, the rods are excited by the turbulence in the jet flow as confirmed by the wide band power spectral density (PSD) plot (see Fig. 8a). The flow velocity $V_{Jet}$ = 1.5 m/s marks the stability boundary for the rod bundle. Above the critical velocity (1.5 m/s), the response amplitude is increased sharply. This corresponds to the phenomenon of fluidelastic instability (FEI), where rods vibrate sinusoidally at the single natural frequency of the rod bundle as shown in Fig. 8b. The maximum vibration amplitude in the first two rows reaches up to 30% of the gap within a jet velocity interval of 0.25 m/s. While the third row response is decreased to 18%gap. The downstream rows are less affected by the jet flow due to the spreading and mixing rate of the jet.

### Jet cross-flow induced vibrations of rod bundle for 10-fin shark-inspired nozzle

**Figure 9:**
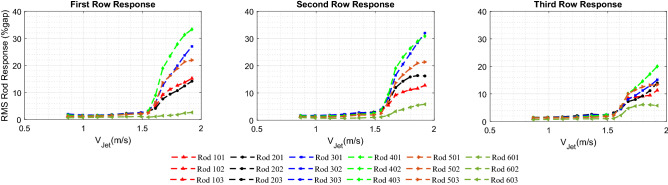
Rod bundle vibrations using 10 fins shark-inspired nozzle.

The rod bundle vibrations with the circular jet flow provides a reference basis for the performance evaluation of the shark-inspired nozzles. The biomimicry design is investigated first with the 10 fins shark-inspired nozzle. Fluidelastic behavior of the rod bundle is obtained by increasing the jet velocity from 0.87 m/s to 1.92 m/s. Figure 9 presents the rod bundle vibration results for the biomimetic nozzle. The attached fins showed their influence by delaying the critical velocity from 1.5 m/s in the circular nozzle case to 1.55 m/s. Furthermore, the maximum response in the first row (rod 401) is reduced by 10% while the reduction percentage is increased 20% and 26% for second row and third row rods, respectively. The interesting results of the 10 fins shark-inspired nozzle on mitigation of jet cross-flow induced vibrations lead us to investigate another biomimetic nozzle with higher number of fins to show its effect on the mixing process.

**Figure 10:**
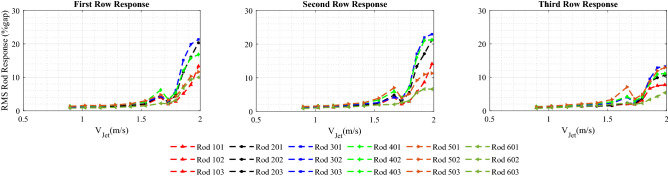
Rod bundle vibrations using 15 fins shark-inspired nozzle.

### Jet cross-flow induced vibrations in rod bundle due to the shark-inspired nozzle with 15 fins

**Figure 11:**
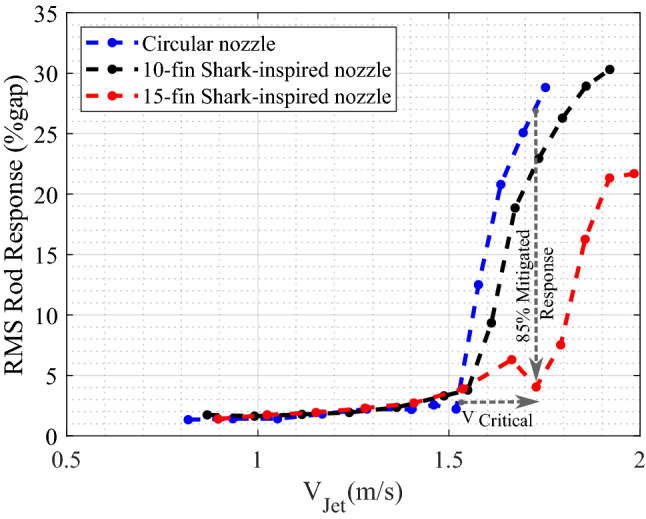
Comparison of rod 402 response with three different nozzles.

A second shark-inspired nozzle with 15 fins is tested to investigate the effect of the number of flow channels on the rod array vibration, increasing number of fins corresponds increasing the mixing rate of the jet flow. Figure 10 shows the RMS rod response obtained with 15 fins shark-inspired nozzle. As seen from this figure, the fin number has a major impact on the dynamical behavior of the rod bundle. The jet velocity at which instability occurred is significantly delayed, from 1.5 m/s to 1.75 m/s. Compared with the test conducted with the circular jet, the maximum response in the first row (rod 401) is mitigated by 85%, and the mitigation percentage for the second row (rod 402) response is almost the same at 81% while, this percentage became 75% for the third row response (rod 403). Furthermore, unlike with the circular jet cross-flow induced vibrations, the increasing rate in the unstable vibration response is not as sharp as shown in Fig. 11. Compared with the results obtained with the 10 fins shark-inspired nozzle, rod 401 vibration is mitigated by 53%, while the vibration of rods 402 and 403 is eliminated by 32% and 46%, respectively. Consequently, the comparison between the results obtained with the three nozzle designs lead to the conclusion that the biomimetic nozzle design has a significant effect on the mixing rate of the jet flow. This results in a major reduction in the rod bundle vibration.

In addition, the number of fins is important parameter when considering the mixing rate and water flow resistance through the nozzle. Increasing the number of fins shows an interesting effect on the percentage reduction in the rod bundle vibrations. However the reduction in effective nozzle diameter should be carefully considered as fin number increases due to associated increase in pressure drop. The 10 fins shark-inspired nozzle reduces the base diameter by 3.5% due to the fins volume. Moreover, this reduction in diameter increases to 5% for the 15 fin nozzle. However, the results with the 15 fins shark-inspired nozzle show a significant mixing efficiency compared to the other tested nozzles. Thus, the mixing rate and pressure drop across the biomimetic nozzle with 15 fins is optimal.

## Discussion

**Figure 12:**
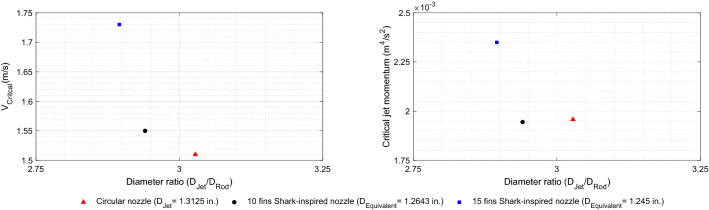
Comparison of critical jet velocity and momentum from the three tested nozzles.

In this paper, a new biomimetic nozzle based on the shark gill slits is designed to mitigate the jet cross-flow induced vibrations by providing efficient and rapid mixing between the jet flow and surrounding fluid. The effect of the proposed shark-inspired nozzle is evaluated versus the circular nozzle in the rod bundle vibrations. The experimental results show a significant vibration amplitude reduction (85%) obtained by replacing the circular nozzle with the shark-inspired nozzle. An early study^19^ that identified the planar jet flow induced instability showed that the instability threshold is quantified by the jet momentum. Thus, the reduction in the area due to the attached fins should be considered together with jet critical velocity by introducing the critical jet momentum. Equation (1) is used to calculate the jet momentum at the stability threshold.

1 $$\begin{aligned} {M_{Critical}}={A_{Exit}}{V_{Critical}^2} \end{aligned}$$

where $M_{Critical}$ is the critical jet momentum, $A_{Exit}$ the cross-sectional area of the nozzle, and $V_{Critical}$ the jet velocity at the instability threshold. Figure 12 shows the critical jet velocities and the corresponding jet critical momentum plotted versus the diameter ratio between the nozzle and the rod. For the shark-inspired nozzles, an equivalent diameter is calculated from the exit cross-sectional area of the nozzle. The stabilizing effect of the 10 fin shark-inspired nozzle may come from the blockage effect of the fins, as a result of equal momentum value with that obtained with the circular nozzle. However, increasing the number of fins to 15 affects the fluid dynamics of the jet flow; the critical momentum is increased by 20% meaning that the rod bundle is safe from instability with this biomimetic nozzle. Increasing the fin number channels the main flow into more streams which appears to enhances mixing.

## Conclusions

This study is conducted to propose a solution to grid-to-rod fretting of fuel assemblies proximity to the loss-of-coolant accident (LOCA) holes that has been observed in some nuclear reactors. The injected flow from LOCA holes causes fuel rod vibration, which can lead to wear and fretting with their supports (spacer grids). The most effective way to reduce vibration caused by jet cross flow is to improve mixing between the jet potential core and the surrounding fluid, which causes the jet velocity to decay faster and its impact on the rods to be reduced.

Biomimicry of shark gill slits configuration can be incorporated to design a biomimetic nozzle by attaching very thin fins mimicking the shark’s secondary lamellae. After the gas exchange process, the exit flow behaviour from the gill slits is of interest due to its steadiness and smoothness. Following the biomimciry design approach that proven through experiments on the rod bundle vibration with two shark-mimetic nozzles; 10 and 15 fins, we found that:

(i) The shark-inspired nozzle has a significant effect on the vibration amplitude and the stability threshold of the jet cross-flow induced vibrations. The minimum vibration response was obtained with 15 fins which is consistent with very confined flow channels of the shark’s secondary lamellae. However, the blockage effect of fins should be carefully considered with increasing the fin number.
(ii) The 15 fin shark-inspired nozzle increased the critical jet velocity from 1.5 m/s to 1.75 m/s in the case of the circular nozzle, which mitigates the rod bundle vibration by 85%. This vibration amplitude reduction can contribute to an increased the safety limit for reactors during normal operation.
(iii) FEI occurs when the jet momentum exceeds the critical limit. Experiments with the 15 fin shark-inspired nozzle confirmed that the critical jet momentum is increased by 20% when the circular nozzle is replaced by proposed biomimetic nozzle.

Highly efficient mixing nozzles are of great interest for a variety of applications. The shark-inspired nozzles can be integrated into fuel injection systems, atomizers and gas mixers to improve the mixing rate. The number of the attached fins will directly affect the jet flow dynamics and mixing efficiency. The fin number and pressure drop around the nozzle must be optimised.

## Data Availability

The datasets generated and/or analysed during the current study are not publicly available due to inclusion of proprietary information on industrial application (in nuclear engineering) and pending patent application, but are available from the corresponding author on reasonable request.

## References

[1] Crow SC, Champagne F (1971). Orderly structure in jet turbulence. J. Fluid Mech..

[2] Yule A (1978). Large-scale structure in the mixing layer of a round jet. J. Fluid Mech..

[3] Gutmark E, Ho C-M (1983). Preferred modes and the spreading rates of jets. Phys. Fluids.

[4] Ho C-M, Huerre P (1984). Perturbed free shear layers. Annu. Rev. Fluid Mech..

[5] Becker HA, Massaro T (1968). Vortex evolution in a round jet. J. Fluid Mech..

[6] Zaman, K. B., Samimy, M. & Reeder, M. Effect of tabs on the evolution of an axisymmetric jet. In *Symposium on Turbulent Shear Flows*, NASA-TM-104472 (1991).

[7] Reeder M, Samimy M (1996). The evolution of a jet with vortex-generating tabs: real-time visualization and quantitative measurements. J. Fluid Mech..

[8] Phanindra BC, Rathakrishnan E (2010). Corrugated tabs for supersonic jet control. AIAA J..

[9] Krishnaraj A, Ganesan V (2021). Investigation of jet mixing characteristics using slotted rectangular tabs. J. Aerosp. Technol. Manage..

[10] Thangaraj T, Kaushik M, Deb D, Unguresan M, Muresan V (2022). Survey on vortex shedding tabs as supersonic jet control. Front. Phys..

[11] International Atomic Energy Agency. Review of fuel failures in water cooled reactors. https://www.iaea.org/publications/8259/review-of-fuel-failures-in-water-cooled-reactors (2015).

[12] United states nuclear regulatory commission office of inspection and enforcement. Fuel pin damage due to water jet from baffle plate corner. https://www.nrc.gov/reading-rm/doc-collections/gen-comm/circulars/1980/cr80017.html (1980).

[13] Bischof, G.T. Report No. 50-339/2014-002-00, Virginia Electric and Power Company, North Anna Power Station. https://www.nrc.gov/docs/ML1432/ML14325A692.pdf (2014).

[14] Miklosovic D, Murray M, Howle L, Fish F (2004). Leading-edge tubercles delay stall on humpback whale (megaptera novaeangliae) flippers. Phys. Fluids.

[15] Gad-el Hak I (2019). Fluid-structure interaction for biomimetic design of an innovative lightweight turboexpander. Biomimetics.

[16] Muir B, Kendall J (1968). Structural modifications in the gills of tunas and some other oceanic fishes. Copeia.

[17] Hughes G (1966). The dimensions of fish gills in relation to their function. J. Exp. Biol..

[18] Benz GW (1984). On the conservative nature of the gill filaments of sharks. Environ. Biol. Fishes.

[19] Fujita K, Ito T, Kohno N (1990). Experimental study on the vibration of circular cylinders subjected to cross-flow jetted from a narrow gap. J. Fluids Struct..

